# Improving care for sexually transmitted infections

**DOI:** 10.1002/jia2.25349

**Published:** 2019-08-30

**Authors:** Cornelis A Rietmeijer

**Affiliations:** ^1^ Rietmeijer Consulting Denver CO USA; ^2^ Colorado School of Public Health University of Colorado Denver CO USA

**Keywords:** STI, medical care, prevention, differentiated care, HIV prevention, health systems

## Abstract

**Introduction:**

Rising rates of reported sexually transmitted infections (STIs) in the US and Europe are a public health priority and require a public health response. The diagnosis and treatment of STIs have been the cornerstone of STI control and prevention for many decades and, historically, publicly funded STI clinics have played a central role in the provision of STI care. Innovations in non‐invasive diagnostic techniques, especially nucleic acid amplification tests in the mid‐1990s, have facilitated the expansion of STI testing and treatment outside traditional STI clinics, including primary care, family planning, school‐based health, outreach, corrections, emergency departments and HIV prevention and care settings. As a result, the continued need for categorical STI clinics has been debated. In this Commentary, we discuss how practice can be improved at each level of STI care.

**Discussion:**

STI practice improvement plans should be tailored to the strengths of each care setting. Thus, in primary care, the focus should be on improving STI screening rates, the provision of hepatitis B and human papillomavirus vaccines and, in jurisdictions where this is legal, expedited partner therapy for gonorrhoea and chlamydia. Extragenital (pharyngeal and rectal) testing for gonorrhoea and chlamydia should be available in settings serving populations more vulnerable to STI acquisition at these anatomical sites, including men who have sex with men. In family planning settings with a mostly female patient population, there are opportunities to serve male partners with both contraceptive and STI services. STI screening rates can also be improved in other settings serving populations at increased risk for STIs, including school‐based clinics, emergency departments, correctional health facilities and providers of HIV care and prevention. These improvements are predominantly logistical in nature and not dependent on extensive STI clinical expertise. While some providers in these settings may have the clinical knowledge and skills to evaluate symptomatic patients, many do not, and STI speciality clinics must be available for consultation and referral and evolve from “safety net” providers of last resort to STI centres of excellence.

**Conclusions:**

A tailored practice improvement plan can be envisioned to achieve an optimally functioning STI care continuum.

## 1 Introduction

The consistent rise in the number of reported sexually transmitted infections (STIs) in the US 1 and Europe 2 presents a public health priority requiring an urgent public health response.

The reasons for rising STI rates are not fully understood. Men who have sex with men (MSM) are most vulnerable to STI acquisition and have experienced disproportional increases in gonorrhoea and syphilis rates 1. Evidence suggests that changing perspectives on HIV transmission risks brought about by effective HIV treatment and pre‐exposure prophylaxis (PrEP) have led to changes in attitudes towards condom use and other prevention strategies with the unfortunate result that HIV risk reduction may be accompanied by increasing the risks for other STIs 3, 4, 5. However, rising STI rates are not limited to MSM. The resurgence of syphilis in the US now also involves heterosexual men and women, and the increasing congenital syphilis rates are alarming 1. Other reasons may contribute to rising STI rates. Substance use (“chemsex”) is associated with increased sexual risk behaviours 6, 7 and the recruitment of sex partners is facilitated by online dating sites and apps 8. Increased case finding also plays a role, for example, the implementation and adherence to annual chlamydia screening for sexually active women 9. In addition, it has been appreciated for some 15 years that asymptomatic extragenital (pharyngeal and rectal) gonorrhoea and chlamydia infections are very common among MSM and that failure to screen these anatomical sites may lead to underestimating the infection burden by more than 50% 10. Current STI screening guidelines stress the importance of extragenital testing among MSM 9, and thus lead to enhanced case finding. Finally, a fraying public health infrastructure is blamed for the syphilis resurgence among heterosexual populations and the associated rise in congenital syphilis 1.

While the underlying causes of the rising STI trends will continue to be elucidated, this should not delay an urgently needed public health response.

Historically, the diagnosis and treatment of STIs have played a key role in public health STI control efforts. However, while the concept of “treatment as prevention” has only recently entered the lexicon of HIV prevention 11, it has been the guiding principle for STI control and prevention for many decades, enabled by the introduction of penicillin and other antibiotics after the second world war when syphilis and gonorrhoea were at epidemic highs. Given the public health importance of STI treatment and the stigma associated with these diseases, publicly funded “categorical” STI clinics became a critical component in the fight against STIs. Frequented by patients with symptomatic STIs who did not have other sources of medical care or who chose these clinics for confidentiality reasons even if they had access to other care providers, these clinics became a “safety net” for stigmatized populations at high risk for STIs, including MSM, sex workers and people who inject drugs.

An important limitation of relying on the care of symptomatic patients to control STI was the increasing recognition of the asymptomatic nature of many STIs and a growing awareness that STI control could not be accomplished by just focusing on patients with symptomatic infections: the proverbial tip of the iceberg. However, the alternative – the establishment of screening programmes for asymptomatic (high‐risk) persons – was stymied by insensitive and cumbersome tests requiring invasive (urethral, cervical) sampling techniques that were not widely available and not particularly attractive to the public.

The development of highly sensitive nucleic acid amplification tests (NAATs) using non‐invasive, self‐sampled specimens (urine, vaginal or anal swabs) have dramatically changed the STI prevention landscape since the mid‐1990s 12. Such tests, including combined chlamydia/gonorrhoea NAATs, could now be done easily in a variety of non‐STI clinic settings, including primary care, family planning, HIV prevention and care and even outreach 13 as well as home‐based testing programmes facilitated by the growing popularity of the Internet 14. Public health screening recommendations, for example, routine annual chlamydia screening for young sexually active women 9, became feasible. As a result, increasing numbers of STIs, especially chlamydia infections, are now reported from non‐STI clinic settings, including primary care (both private and public) and family planning clinics 1.

With the widening array of STI care providers and with increasing access to these providers, for example, through the implementation of the Affordable Care Act in the US, the role of publicly funded STI clinics as safety net providers has become increasingly scrutinized and a number of clinics have closed their doors or have curtailed their services 15. Unfortunately, at the same time, STI rates have been increasing in the US and elsewhere, and it is tempting to speculate that the dismantling of the public health STI care infrastructure may be causally related to these trends 15.

## 2 Discussion: Improving STI services

The increasing importance of multiple sources in the overall provision of STI care should be recognized. Rather than fearing a fragmented system, a practice improvement plan should be designed that builds on this diversity and tailors recommendations to the STI services that are provided at each level.

### 2.1 Primary care

Screening for chlamydia and gonorrhoea using non‐invasive NAATs has become a standard of practice in many primary care settings, including private providers and publicly funded health centres. Indeed, a large number of infections are reported from these providers already 1. But there is room for improvement. It is estimated that only 40% to 50% of sexually active women under the age of 25 are screened for chlamydia annually in primary care settings in the US 16. With advances in electronic medical records, allowing for automated prompts, as well as test reimbursement schemes, there is no reason why screening rates should not be higher.

Likewise, coverage for HBV and HPV vaccinations can be improved by including it in standard immunization schemes recommended for primary care settings 17. Also, in jurisdictions where this is legal, primary care providers should be encouraged to implement expedited partner treatment (EPT) for patients diagnosed with gonorrhoea or chlamydia 9.

However, while some primary care physicians serve populations at high risk for STIs and are quite comfortable with the differential diagnosis and treatment of STI, most encounter symptomatic STIs infrequently, and their expertise may vary when evaluating and treating patients presenting with relatively rare STI, including primary and secondary syphilis and lymphogranuloma venereum. Developing such skills would not be practical in settings with an already overburdened medical staff. It is important, however, that they should have easy access to consultation with STI experts in their region or through online resources 18.

### 2.2 Family planning

Priorities in family planning facilities are focused on the provision of contraception, but with growing expertise, these clinics have become important providers of STI care, especially for women. Screening for chlamydia and other STIs has become common practice in this setting, especially since the widespread adoption of chlamydia/gonorrhoea NAAT assays. Family planning clinics are also increasingly encouraged to expand their services to men. However, even though average male attendance is growing, it is still low in many clinics, for example, less than 10% in publicly funded family planning clinics in the US 19. As a more holistic sexual health paradigm is gaining ground 20, further STI service and skills development in family planning clinics and appeal to other populations would be a welcomed expansion of the STI care infrastructure.

### 2.3 HIV prevention and care settings

The resurgence of STIs among MSM 3 has profoundly affected traditional HIV prevention and care settings. HIV testing sites, whether clinic‐ or outreach‐based, are increasingly providing chlamydia/gonorrhoea NAATs and syphilis serologic testing. Many sites now offer chlamydia/gonorrhoea testing for all exposed anatomical sites (including urine, anal and pharyngeal sampling) and, with most laboratories validated for testing of extragenital samples, this should be the standard of care at these settings. However, many HIV testing sites are staffed by non‐medical (outreach) providers, and clinical expertise is often not available for further evaluation and treatment. Strong collaborations with local STI or HIV care clinics are necessary for consultation, treatment and follow up of clients presenting with (symptomatic) STIs 21.

Persons living with HIV, especially MSM, are at disproportionate risk for STIs, including syphilis, gonorrhoea and chlamydia 1. Regular screening for these infections, including extragenital gonorrhoea/chlamydia testing, should thus be the standard of care in HIV care practice. Most guidelines recommend screening at six‐month intervals, but the frequency should be determined by sexual risk assessment 9. Since HIV care providers (in contrast to STI clinics) see their patients regularly, they have a unique opportunity to identify and treat incident STIs in this key population.

Models for the provision of HIV PrEP are developing, ranging from active referral mechanisms to on site provision of antiretrovirals in a variety of settings, such as HIV care, STI clinics and primary care. There is much debate about whether PrEP is related to increases in sexual risk behaviours. But there is no doubt that persons on PrEP have a high risk for STIs 22 and regular (three to six months) screening for STIs should thus be part of the standard of PrEP care 23.

### 2.4 Other settings

Given the highest rates of chlamydia and gonorrhoea among women aged 15 to 20 years and men aged 20 to 25 1, there is a strong rationale for offering basic STI services, including chlamydia and gonorrhoea screening and condom distribution to sexually active adolescents and young adults in school‐ and college‐based health centres. At least one recent US study suggests that there is considerable public support for offering these services in these settings 24. Other settings serving populations at high risk for STIs where basic STI screening is feasible but not yet fully scaled up include correctional facilities 25, 26 and emergency departments 27.

### 2.5 The future of the STI clinic

Within the landscape of multiple STI care providers, evidence supports the continued importance of categorical STI clinics. In numerous countries where health insurance is near universal and where primary healthcare providers offer basic STI testing, STI clinics are nonetheless thriving. For example, the STI clinic in Amsterdam is on course to see almost twice the number of patients in 2018 (50,000 visits) than it saw in 2000. This is despite universal healthcare access in the Netherlands and a clinic policy that defers low‐risk and asymptomatic patients to their primary care physicians.

This growth in patient population is in large part but not exclusively due to increasing numbers of MSM visiting the clinic, reflective of higher rates of STI in this population over the past two decades 28. Similar shifts towards higher proportions of MSM visiting STI clinics has been observed elsewhere, including the US 29, 30. Reasons for continued use of STI clinics include client perceptions of clinic expertise, confidentiality, easy access, same‐day services and low or no cost 31. Even patients with newly acquired health insurance will continue to use the STI clinic as they may be reluctant to use their insurance due to confidentiality 31.

In this emerging landscape of STI care, what should the future role of publicly funded STI clinics be? Foremost, it should be recognized that categorical STI clinics, unlike other STI service providers, have STI treatment and prevention as their primary public health mission. They should thus function as a central hub in their local and/or regional STI provider network and be an essential partner in the overall STI public health response in the region. Rather than “safety net clinics” that are doomed to become obsolete once access to (primary) health services is assured, these clinics should be centres of excellence that provide the delivery of expert STI clinical care, state‐of‐the‐art diagnostic capabilities and on‐site treatment and follow up, (including EPT). They should be available for low‐threshold referral and consultation. They should also be a resource for sentinel surveillance research, including gonococcal resistance 29, 32, and for research in the development of new STI diagnostics and treatment, as well as for clinical training and workforce development 33, 34.

From a morbidity/mortality and cost perspective, HIV is still the most important STI. STI clinics disproportionally serve populations at high risk for HIV, diagnose persons with HIV and link them to care, and are becoming an increasingly important gateway for PrEP care 35. HIV prevention services are thus a central component of the STI clinic mission. In fact, some clinics, where patients find it difficult to follow through on HIV care or PrEP referral, have started to provide HIV and PrEP care on site, essentially making the concept of “safety net provider” come full circle 36.

With typically constrained resources, STI clinics must provide their services in the most cost‐efficient manner. Non‐invasive NAATs for the diagnosis of gonorrhoea and chlamydia allow the triage of patients into those that need full examination versus those who need only screening: so‐called “express visits,” which has significantly increased efficiency and lowered costs for STI clinics 37, 38, 39. The “express visit” model has now been widely adopted and has even led to the emergence of stand‐alone express clinics, for example, Dean Street Express in London 40. While such stand‐alone clinics are promising for asymptomatic populations that require frequent STI testing (such as persons receiving HIV PrEP), they may not be staffed to serve patients with symptomatic STI and should thus have a mechanism to refer those patients to STI speciality care 41.

Finally, in an era of dwindling public spending, publicly funded STI clinics should be proactive in finding ways to diversify their funding. Given overlaps between STI and pregnancy risk among (young) women, the provision of family planning services in STI clinics makes sense from a sexual health perspective, and many clinics have integrated these services and broadened their funding base 42.

Billing patients for services may seem to be anathema to the public health mission of STI clinics as it could raise barriers to access. However, carefully designed schemes that encourage patients to use their insurance, while readily allowing them access if they choose not to use insurance and have no other means of paying, could still result in a sizeable source of revenue 43. In the US, nurse practitioners, but not regular nurses, can independently bill for services. This has been an additional impetus for certain clinics to provide a billable service that can be provided by these practitioners, including PrEP and the placement of intrauterine birth control devices and other long‐acting, reversible contraceptives.

Given their patient/client base, STI clinics are also in a good position to apply for (sentinel) surveillance and research projects, including studies on gonococcal antimicrobial resistance and rapid, point‐of‐care diagnostics. Currently, few STI clinics are positioned to profit from these opportunities. However, there are many more clinics that, with additional effort, could rise to a level that would benefit not only their patients but also their bottom line.

## 3 Conclusions

The future of STI control and prevention is daunting, but it is also promising. There is now a large and potentially growing array of STI service providers, both in public and private sectors, that can have significant impact on STI control when forged together in a single vision. The diversity of STI care providers has in large part been made possible by the advent of non‐invasive testing technologies. Further advancement in technology, specifically the development of rapid, sensitive and specific point‐of‐care testing, which is already on the horizon, will provide additional tools for STI diagnosis and control. What is needed above all is a continued passion and advocacy for STI and HIV prevention.

## Competing interests

Dr Rietmeijer is an independent STI consultant. He declares no commercial or other conflicts of interest with the contents of this Commentary and did not receive funding for its writing.

## Authors’ contributions

CAR wrote this manuscript.
